# Technical Quality and Diagnostic Impact of Chest X-rays in Tuberculosis Screening: Insights From a Saudi Teleradiology Cohort

**DOI:** 10.7759/cureus.53509

**Published:** 2024-02-03

**Authors:** Amr M Ajlan

**Affiliations:** 1 Radiology Department, King Abdulaziz University Faculty of Medicine, Jeddah, SAU; 2 Radiology Department, Diagnostics Elite Teleradiology, Jeddah, SAU

**Keywords:** pulmonology, screening, saudi arabia, chest x-ray, tuberculosis

## Abstract

Objectives

To assess the standard of chest X-ray techniques in tuberculosis (TB) screening within Saudi Arabian healthcare facilities and evaluate the impact of technical quality on radiological interpretation.

Materials and methods

Analysis of 250 posteroanterior chest radiographs sourced from a network of five clinics was conducted. These images were scrutinized for technical quality by a radiologist.

Results

Of the radiographs analyzed, 57% exhibited technical issues, with overexposure and clothing artifacts being the most commonly encountered. Notably, only 14% of these radiographs were deemed to have compromised diagnostic ability.

Conclusion

The presence of technical issues in most chest X-rays for TB screening highlights a significant area for improvement. However, the relatively low percentage of radiographs impacting diagnostic quality indicates that most issues do not critically hinder the radiologist's interpretative capability. This underscores the importance of balanced quality control measures in radiographic practices for effective TB detection in the region.

## Introduction

Tuberculosis (TB) remains a significant global health concern, marked by its high incidence and mortality rates. Recent years have seen a worrying decline in TB detection, coupled with an increase in TB-related deaths, attributed partly to limited access to diagnostic and treatment services. Complicating the landscape further is the emergence of drug-resistant TB strains, including multi-drug-resistant and extensively drug-resistant varieties. These strains present formidable challenges in the ongoing battle against TB, underscoring the need for effective diagnostic strategies such as chest X-ray screening, especially in regions like Saudi Arabia [1-4].

Given that TB can go undetected for days and weeks after the onset of symptoms, timely diagnosis in an ever-growing migrant population in Saudi becomes a primary healthcare priority [3,4]. Amongst the several available tests that aid in early screening for TB, chest X-ray remains a commonly utilized modality [5,6]. When combined with the clinical picture, radiographic chest assessment has high sensitivity in detecting TB [6,7]. Thus, it is standard practice to utilize chest X-rays as part of the formal TB screening for new immigrant workers across various primary healthcare facilities in Saudi Arabia and the Gulf region [8,9].

In this study, we investigate a previously unexplored area: the adherence to standard techniques in acquiring chest X-rays across various healthcare facilities for TB screening. Leveraging a teleradiology network that collates TB screening chest X-rays from numerous primary healthcare clinics across different Saudi Arabian regions, our objective is to evaluate the technical quality of these radiographs. Specifically, we aim to determine whether technical suboptimalities in these X-rays impact the radiologist's diagnostic capabilities in interpreting these images for TB screening purposes. This assessment can potentially enhance the effectiveness of TB detection strategies in the region.

## Materials and methods

**Figure 1:**
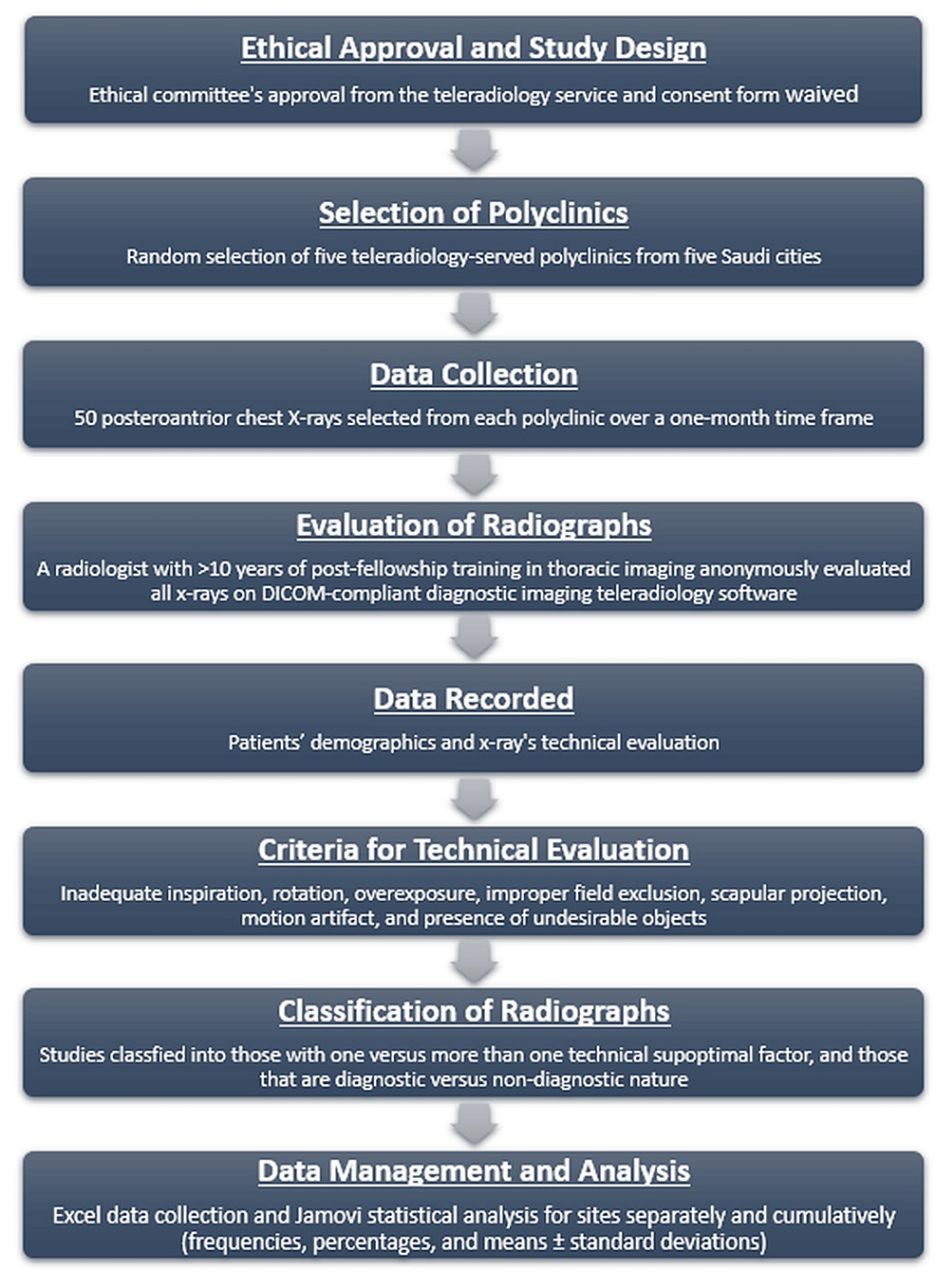
Flow chart representation of the study’s methodology. DICOM: Digital Imaging and Communications in Medicine.

Figure 1 shows a flow chart representation of the study’s methodology. The teleradiology service’s ethical committee board approved the study, waiving the informed consent form. Five teleradiology-served polyclinics were randomly selected for the study from the following Saudi Arabian cities: Jeddah, Abha, Unaizah, Khamis Mushait, and Dammam. For each polyclinic, 50 TB screening posteroanterior (PA) projection chest radiographs were randomly selected for analysis for one month. All examinations were anonymously evaluated by a radiology consultant with more than ten years of post-fellowship training thoracic imaging experience. The examinations were reviewed on a fully web-based Digital Imaging and Communications in Medicine (DICOM)-compliant diagnostic imaging teleradiology software (Paxera Ultima 360, viewer version 6.0.2.6, HTSI Healtcare Solutions, Pembroke Pines, FL, USA).

Patients’ gender and age were recorded. A chest radiograph was evaluated for several technical parameters. Inadequate inspiration was designated if less than nine right posterior ribs were counted above the edge of the right hemidiaphragm. The patient was considered rotated if the distance from the first thoracic vertebral body spinous process to each medial aspect of the clavicular heads was subjectively unequal on the assist radiograph. Radiographic overexposure was defined as the ability to visualize the intervertebral discs behind the mediastinal spine, leading to partial or complete lung darkness persisting even after image contrast manipulation. Improper exclusion of part of the field of view was defined as excluding the apex, base, or lateral edge of one or both lungs. Improper scapular projection over the lung field was defined as visualizing more than one-third of the scapular body projecting over one or both lung fields. Motion artifact was recorded if the diaphragm or lung vasculature were ill-defined due to motion. The reader is also evaluated for projecting undesirable objects on the radiograph, namely hair, metals, and clothing. Objects related to clothing, even those metallic, were included in the clothing category. Radiographs revealing more than one technically suboptimal factor were also recorded. The radiologist also assessed whether the chest radiograph was diagnostic or not from a technical point of view based on his subjective experience.

A collection was recorded in Excel, and statistical analyses were performed with the open-source statistical software Jamovi (Version 2.3.18.0, The Jamovi Project, retrieved from https://www.jamovi.org). Each polyclinic site was analyzed separately, and a final cumulative assessment for all sites was also performed. Categorical variables are frequencies and percentages, and continuous variables are represented as means ± standard deviations (SDs).

## Results

Table 1 lists the information for patients’ gender, patients’ age, and results of technical analysis of the chest radiographs.

**Table 1:** Information for patients’ gender, patients’ age, and results of technical analysis of the chest radiographs. All data are presented as absolute numbers (with corresponding percentages), except for the variable "age." The results are expressed in terms of mean years (with corresponding standard deviations).

Site	1	2	3	4	5	Total
Chest X-ray numbers	50 (100%)	50 (100%)	50 (100%)	50 (100%)	50 (100%)	250 (100%)
Gender	39 (78%)	34 (68%)	41 (82%)	41 (82%)	47 (94%)	202 (81%)
Age	30.20 (±7.4)	35.24 (±6.7)	35.04 (±12.3)	31.96 (±8.3)	31.96 (±7.2)	32.88 (±8.7)
Inadequate inspiration	3 (6%)	4 (8%)	8 (16%)	14 (28%)	8 (16%)	37 (15%)
Rotation	0 (0%)	4 (8%)	8 (16%)	17 (34%)	8 (16%)	37 (15%)
Overexposure	0 (0%)	8 (16%)	18 (36%)	39 (78%)	0 (0%)	65 (26%)
Inadequate field of view	2 (4%)	7 (14%)	0 (0%)	5 (10%)	3 (6%)	17 (7%)
Scapula over-projection	0 (0%)	0 (0%)	0 (0%)	0 (0%)	1 (2%)	1 (0.4%)
Motion artifact	4 (8%)	1 (2%)	0 (0%)	0 (0%)	0 (0%)	5 (2%)
Hair artifact	0 (0%)	0 (0%)	0 (0%)	0 (0%)	0 (0%)	0 (0%)
Metallica artifact	0 (0%)	2 (4.00%)	2 (4%)	2 (4%)	0 (0%)	6 (2%)
Clothing artifact	7 (14%)	18 (36%)	2 (4%)	16 (32%)	3 (6%)	46 (18%)
Cases with a technical issue	16 (32%)	33 (66%)	26 (52%)	46 (92%)	21 (42%)	142 (57%)
Cases with multiple technical issues	1 (2%)	8 (16%)	11 (22%)	35 (70%)	2 (4%)	57 (23%)
Non-diagnostic study	0 (0%)	0 (0%)	0 (0%)	34 (68%)	0 (0%)	34 (14%)

The population's age range was 19-76 years (mean and standard deviation of 32.88 and ±8.7), compromised by a predominant male gender (81%). Of 50 cases per polyclinic, recorded technical issues were as low as 16 (32%) cases in one polyclinic and as high as 46 (92%) cases in another.

Out of 142 cases with a technically suboptimal factor, 57 (23%) cases had more than one technical issue, and 34 (24%) cases were considered non-diagnostic. Of 57 cases with more than one technical issue, 33 (58%) were considered non-diagnostic. One out of the 34 non-diagnostic cases (3%) had only one recorded technical issue.

**Figure 2:**
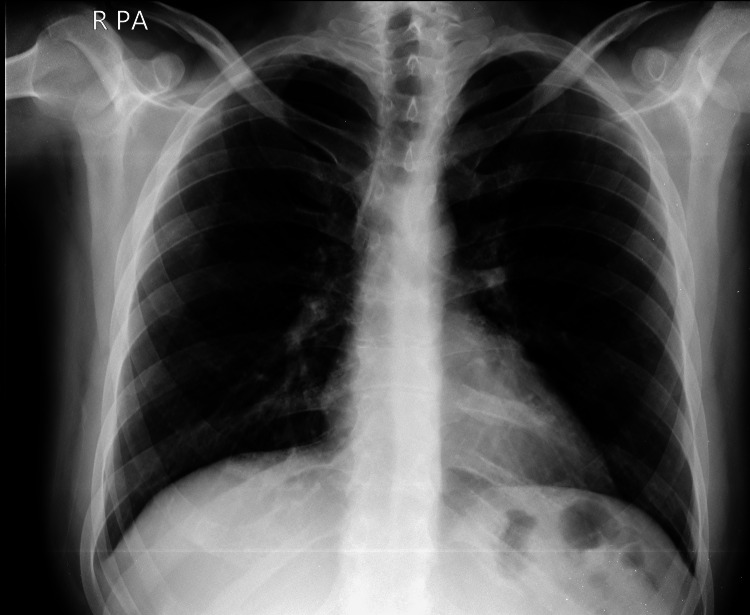
A 32-year-old male with a chest radiograph affected by X-ray overpenetration.

**Figure 3:**
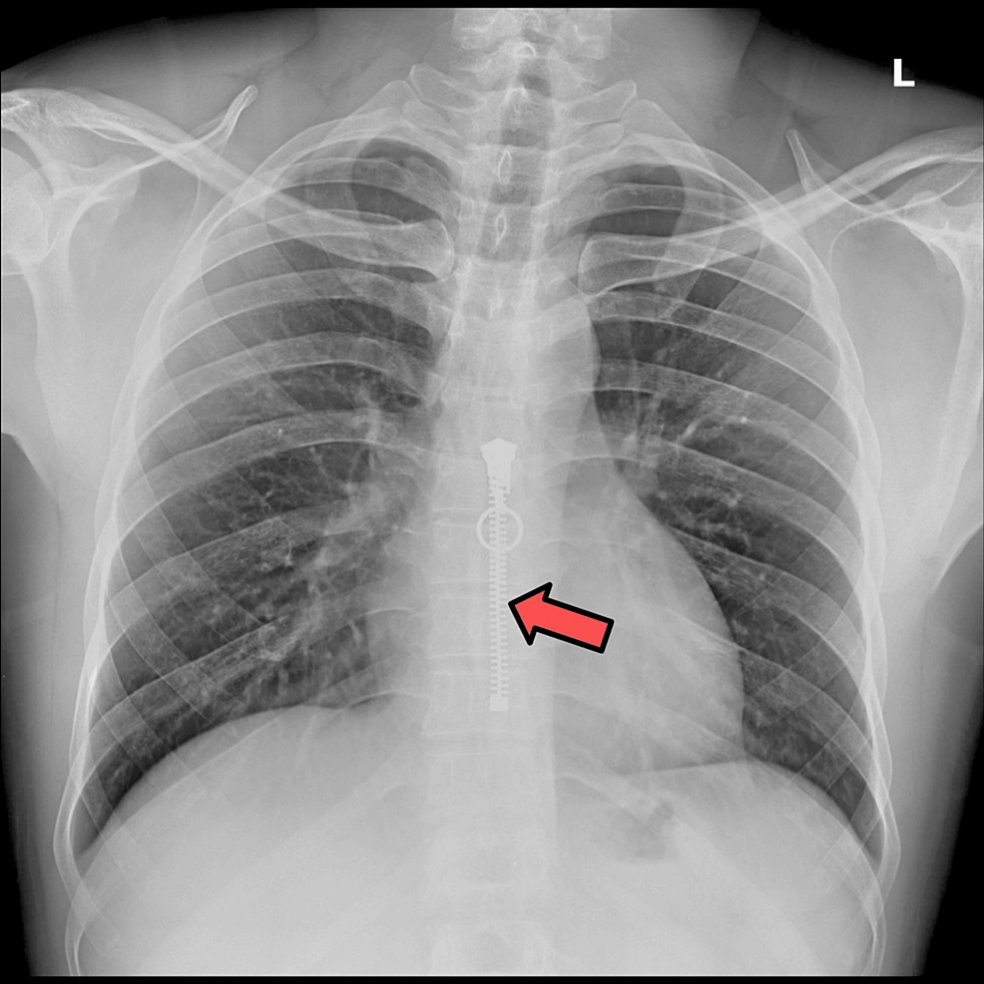
A 31-year-old male with a chest radiograph affected by clothing metallic material (arrow pointing to a zipper).

The study population's most common technical suboptimal factor was x-ray beam overexposure (Figure 2), encountered in 65 (26%) cases. The second most common technical suboptimal factor was metallic and nonmetallic clothing projecting over the lung fields (Figure 3), seen in 46 (18%) cases. Inadequate inspiration and patient rotation were each encountered in 37 (15%) cases.

**Figure 4:**
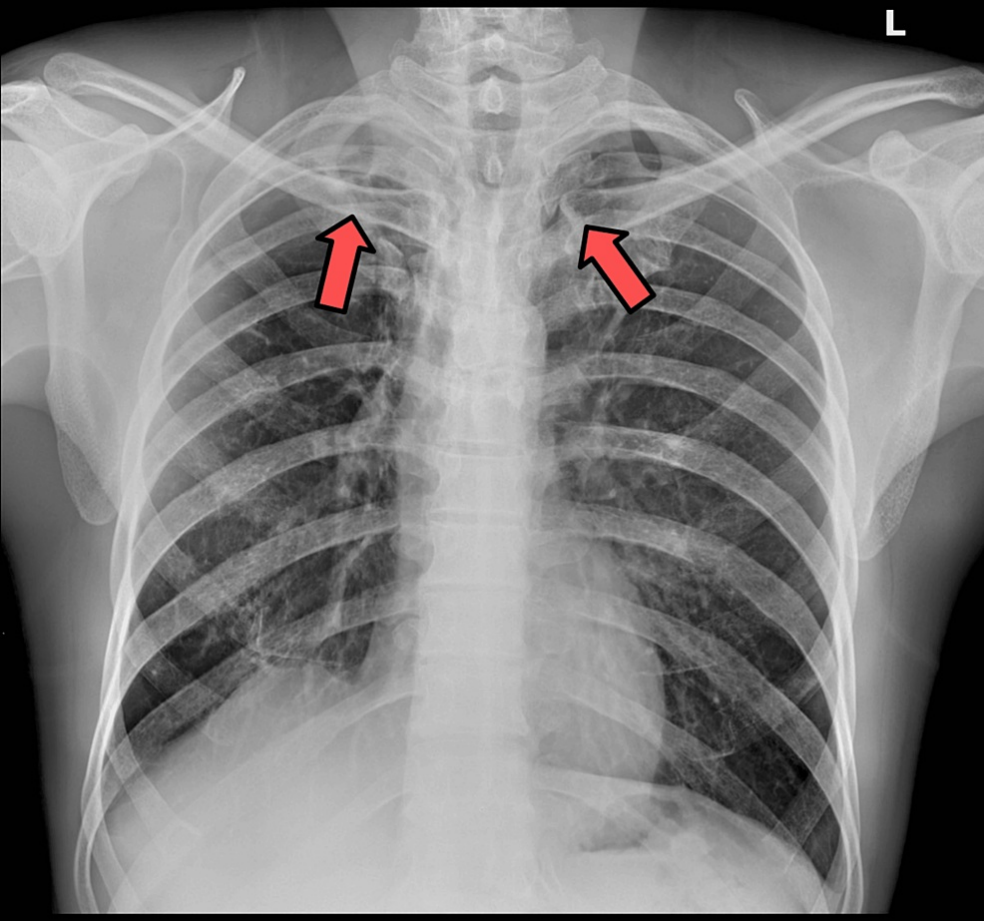
A 50-year-old male with a chest radiograph demonstrating arrows pointing to biapical pleuroparenchymal scarring due to presumed old tuberculosis.

Of 250 cases, 9 (4%) patients had abnormalities on chest radiographs, with 2 (1%) being clinically significant yet not related to findings of active TB. The significant findings were of a case with a 2.8 cm left upper lobe indeterminate nodule and another case with cardiomegaly with pleural effusion in the context of prior coronary arterial bypass grafting. The remainder of the findings were as follows: biapical pleuroparenchymal scarring due to presumed old TB (Figure 4), rib fractures, pneumatocele, left supraspinatus calcific tendinosis, right lower lobe minor plate-like atelectasis, accessory azygos fissure, and an old left clavicle fracture treated by plate and screw fixation.

## Discussion

Saudi Arabia, a destination for a diverse migrant workforce, mandates a PA chest X-ray for both white and blue-collar workers as part of a TB screening protocol. These assessments are conducted across various certified primary healthcare clinics throughout the kingdom. It is generally presumed that these PA chest X-rays adhere to established technical standards [10,11]. However, our study reveals a considerable deviation from this expectation, with 57% of cases exhibiting one or more technical suboptimalities. Consistent with existing literature, the most frequent issues identified were suboptimal exposure, patient rotation [12,13], and a notable presence of clothing artifacts.

Despite the substantial incidence of technical suboptimalities in the chest X-rays studied, only 14% were considered non-diagnostic by the evaluating radiologist. Furthermore, nearly all non-diagnostic cases exhibited multiple technical issues. This finding implies that technical imperfections are common but do not invariably obstruct the radiologist's ability to diagnose TB. The general good health status of the migrant workforce population may influence this outcome. Moreover, the classic radiographic signs of active TB, such as consolidations, cavitations, and pleural effusions, are often distinctly visible on chest X-rays [14,15], facilitating diagnosis despite technical limitations. In our study, incidental findings unrelated to active TB have been only noted in 4% of the cases, with only 1% being clinically relevant.

This study is subject to several limitations. Firstly, the studied sample, although reasonable, may not fully capture the diversity of the clinical spectrum across Saudi Arabia, limiting the generalizability of our findings. Additionally, the retrospective design of our study introduces potential biases, possibly affecting the accuracy and completeness of the information. The focus on technical aspects of radiographs without incorporating clinical outcomes or detailed patient data limits the understanding of the impact of technical quality on TB diagnosis. Furthermore, the subjective nature of radiographic interpretation could lead to variability in recording the findings. Technological variations and standardization issues across clinics, including differences in X-ray machines and exposure techniques, further complicate the consistency of radiographic interpretation. The exclusion of other diagnostic modalities for TB, like sputum tests or molecular assays, limits the scope of our study, as these could provide a more comprehensive picture of TB diagnosis efficiency in conjunction with radiographs. Finally, the applicability of our findings to populations outside of the migrant workforce in Saudi Arabia, considering factors such as age, gender, and underlying health conditions, remains uncertain, affecting our results' generalizability.

## Conclusions

In conclusion, our study underscores the prevalent technical issues in chest X-ray practices in Saudi Arabia, with more than half of the radiographs demonstrating some technical suboptimality. However, only a minority of these technically suboptimal radiographs impacted the diagnostic ability for tuberculosis (TB). These findings highlight the resilience of radiographic interpretation despite technical shortcomings. This resilience may be attributed to the generally healthy migrant workforce population and the overt nature of classic TB findings on X-rays. Nevertheless, there is a clear imperative for improved quality control and enhanced training for radiology technicians. Standardizing protocols could significantly elevate the quality of chest X-rays, potentially enhancing TB detection accuracy. Future research should focus on longitudinal studies to monitor quality improvements over time and explore comparative efficacy with other TB diagnostic methods. These efforts are valuable for refining TB screening strategies in the region.

## References

[1] Huang Y, Ai L, Wang X, Sun Z, Wang F (2022). Review and updates on the diagnosis of tuberculosis. J Clin Med.

[2] Kaufmann SH (2016). EFIS lecture. Immune response to tuberculosis: how to control the most successful pathogen on earth. Immunol Lett.

[3] Al-Orainey I, Alhedaithy MA, Alanazi AR, Barry MA, Almajid FM (2013). Tuberculosis incidence trends in Saudi Arabia over 20 years: 1991-2010. Ann Thorac Med.

[4] Saati AA, Khurram M, Faidah H, Haseeb A, Iriti M (2021). A Saudi Arabian public health perspective of tuberculosis. Int J Environ Res Public Health.

[5] Soares TR, Oliveira RD, Liu YE (2023). Evaluation of chest X-ray with automated interpretation algorithms for mass tuberculosis screening in prisons: a cross-sectional study. Lancet Reg Health Am.

[6] Nalunjogi J, Mugabe F, Najjingo I (2021). Accuracy and incremental yield of the chest X-ray in screening for tuberculosis in uganda: a cross-sectional study. Tuberc Res Treat.

[7] John S, Abdulkarim S, Usman S, Rahman MdT, Creswell J (2023). Comparing tuberculosis symptom screening to chest X-ray with artificial intelligence in an active case finding campaign in Northeast Nigeria. BMC Glob Public Heal.

[8] Singh J, Al-Abri S, Petersen E (2022). Importance of tuberculosis screening of resident visa applicants in low TB incidence settings: experience from Oman. J Epidemiol Glob Health.

[9] Al Jahdali HH, Baharoon S, Abba AA (2010). Saudi guidelines for testing and treatment of latent tuberculosis infection. Ann Saudi Med.

[10] Bansal T, Beese R (2019). Interpreting a chest X-ray. Br J Hosp Med (Lond).

[11] Oura D, Sato S, Honma Y, Kuwajima S, Sugimori H (2023). Quality assurance of chest X-ray images with a combination of deep learning methods. Appl Sci.

[12] Al-Malki MA, Abulfaraj WH, Bhuiyan SI, Kinsara AA (2003). A study on radiographic repeat rate data of several hospitals in Jeddah. Radiat Prot Dosimetry.

[13] da Silva WC, Marques MA, do Nascimento AV (2013). Comparative study to determine technical failures affecting conventional chest radiography (Article in Portuguese). Radiol Bras.

[14] Wetscherek MT, Sadler TJ, Lee JY, Karia S, Babar JL (2022). Active pulmonary tuberculosis: something old, something new, something borrowed, something blue. Insights Imaging.

[15] Jeong YJ, Lee KS (2008). Pulmonary tuberculosis: up-to-date imaging and management. AJR Am J Roentgenol.

